# Integrating BRAF^V600E^ mutation, ultrasonic and clinicopathologic characteristics for predicting the risk of cervical central lymph node metastasis in papillary thyroid carcinoma

**DOI:** 10.1186/s12885-022-09550-z

**Published:** 2022-04-27

**Authors:** Zheng Zhang, Xin Zhang, Yifei Yin, Shuangshuang Zhao, Keke Wang, Mengyuan Shang, Baoding Chen, Xincai Wu

**Affiliations:** 1 Department of Medical Ultrasound, Affiliated Hospital of Jiangsu University, Zhenjiang, 212000 People’s Republic of China; 2 Department of Medical Ultrasound, Affiliated Hospital of Nantong University, Nantong, 226006 People’s Republic of China

**Keywords:** Papillary thyroid carcinoma, Cervical central lymph node metastases, Ultrasonic features, Clinicopathologic factors, BRAF^V600E^ mutation

## Abstract

**Background:**

The advantages of prophylactic central lymph node dissection (CLND) for clinically node-negative patients remained a great deal of controversies. Our research was aimed to analyze the relationship between cervical central lymph node metastasis (CLNM) and BRAF^V600E^ mutation, ultrasonic and clinicopathologic characterizes in papillary thyroid carcinoma (PTC).

**Methods and materials:**

In current study, a total of 112 consecutive PTC patients who experienced thyroidectomy plus cervical central neck dissection were included in our research. All PTC were pre-operatively analyzed by ultrasonic features, including tumor size, multifocality or not, tumor location, internal components, echogenicity, microcalcification, margins, orientation, taller than wide shape, and internal vascularity. The presence of clinicopathologic factors, including age, sex, T stage, Hashimoto’s thyroiditis, and BRAF^V600E^ mutation was then investigated. Univariate and multivariate analysis were conducted to check into the relationship between predictive factors and cervical CLNM in PTC patients, and then a predictive model was also established.

**Results:**

Pathologically, 58.0% (65/112) of the PTC patients harbored cervical CLNM. Univariate and multivariate analysis were conducted to identify age < 55 years, tumor size > 10 mm, microcalcification, non-concomitant Hashimoto’s thyroiditis and BRAF^V600E^ mutation were predictive factors for cervical CLNM in PTC. The risk score for cervical CLNM in PTC patients was calculated: risk score = 1.284 × (if age < 55 years) + 1.241 × (if tumor size > 10 mm) + 1.143 × (if microcalcification) – 2.097 × (if concomitant Hashimoto’s thyroiditis) + 1.628 × (if BRAF^V600E^ mutation).

**Conclusion:**

Age < 55 years old, PTC > 10 mm, microcalcification, non-concomitant Hashimoto’s thyroiditis and BRAF^V600E^ mutation are predictive factors for cervical CLNM. BRAF^V600E^ mutation by pre-operative US-FNA technology synergized with clinicopathologic and ultrasonic features is expected to guide the appropriate surgical management for PTC patients.

## Introduction

Papillary thyroid carcinoma (PTC) is the most prevalent histological type of thyroid carcinoma [1], which is largely owing to the extensive utilization of high-resolution ultrasound (US) and fine-needle aspiration (FNA) technology [2–4]. Most of PTC patients require an satisfactory clinical prognosis, after the suitable surgical resection and medical treatment [5]. However, accumulating evidence has documented that 40–60% of PTC cases harbor cervical central lymph node metastasis (CLNM), which could increase the possibility of recurrence, distant metastasis and even contribute to PTC-related deaths [6]. Although development in advance of evidence-based guidelines for PTC management, the advantages of prophylactic central lymph node dissection (CLND) in PTC patients without clinical evidence of cervical CLNM remains a great deal of controversies [7, 8].  Some claim that prophylactic CLND could reduce the recurrence rate of PTC to avoid second dissection, while others argue that patients may not gain any benefit, yet suffer from severe complications, including recurrent laryngeal nerve injury and low parathyroid function [9, 10].

High-frequency US are commonly performed for decision-making in the surgical management of malignant PTC. Solid component, hyper-echogenicity, microcalcification, irregular shapes, poorly defined margins and taller-than-wide shapes are malignant US characterizes that are helpful to judge the malignancy of PTC [11, 12]. Unfortunately, it is challenging to search cervical CLNM attributed to the gas interference in the trachea and esophagus, causing a diagnostic accuracy of merely 30% [13, 14]. US-guided fine-needle aspiration biopsy (FNAB) is widely used to increase the diagnostic accuracy by cytologic and genomic detection. B-type Raf (BRAF) kinase mutation on exon 15 is related to the protein kinase pathway and leads to proliferation and metastasis of PTC [15, 16].  There are still greatly controversies regarding the value of BRAF gene in predicting cervical CLNM [17, 18]. Virk and colleagues have reported PTC patients bearing BRAF mutation were more likely to have cervical CLNM features [19], but one opposite research documented that BRAF mutation was not a risk factor for CLNM in PTC patients [20].

In our study, we also retrospectively reviewed 112 consecutive PTC patients who experienced thyroidectomy plus prophylactic cervical CLND. Univariate and multivariate analysis were also conducted to estimate the relationship between cervical CLNM and BRAF mutation, ultrasonic and clinicopathologic features in patients harboring PTC. Our research is aimed to collaborate BRAF^V600E^ mutation through preoperative US-FNA technology with clinicopathologic and US characteristics for guiding the suitable therapeutic management of PTC.

## Methods and materials

This study design followed the international regulations according to the Declaration of Helsinki. Our research was approved by the Ethical Committee of the Affiliated Hospital of Jiangsu University (SWYXLL20190225-2) and written informed consent was obtained from participants.

### US and clinicopathologic analysis

Between January 2020 and December 2020, a total of 112 consecutive PTC patients who experienced thyroidectomy plus with routinely prophylactic CLND were included in our investigation. All participants had experienced pre-operative US scanning and postoperative pathology to diagnose PTC. The inclusion criteria were retained: (a) participants more than18 years of age; (b) the US images should be retrievable; (c) the US inspections should be conducted within one month before thyroidectomy; (d) PTC with or without cervical LNM should be confirmed by pathological specimens. The exclusion criteria were retained: (a) PTC contained coarse calcifications; (b) US information was insufficient; (c) thyroid nodules were pathologically confirmed as other kinds of thyroid carcinomas. Of note, the largest nodule was picked out into our research when participants with multiple malignant PTCs.

### US evaluation

Participants were required to lie supine with their head slightly bent back. PTC was present in the center of the US screen, and sufficient neighbor thyroid tissue was displayed in the one graph. The targeted PTC was scanned from left to right and from top to bottom. The graphs of the targeted PTC were recorded transversely and longitudinally on gray-scale modality. The US images were interpreted by two experienced radiologists who were unaware of any information about participants' identity and pathological results. The disagreement PTC was re-evaluated and decided by another experienced radiologist. The following US characteristics of targeted PTC were analyzed: diameter on gray-scale US (> 10 mm/ ≤ 10 mm in size), tumor location (left, right and isthmus), multiple PTCs (present/absent), solid (present/absent), marked hypo-echogenicity/hypo-echogenicity (present/absent), microcalcification (present/absent), margin (well defined/poorly defined), shape (regular/irregular), and taller-than-wide shape (present/absent), and internal vascularity (present/absent). We defined microcalcification as calcifications < 1.0 mm in diameter and multifocality as two or more PTCs during US examination.

### Thyroidectomy plus neck dissection

The extent of thyroid surgery was performed based on the American Thyroid Association (ATA) management guidelines [5]. Once nodule malignancy was diagnosed form frozen section, central neck dissection was carefully conducted on the pre-laryngeal and pre-/para-tracheal lymph nodes.

### Histopathologic analysis

Histopathologic results were interpreted by an experienced pathologist. With the utilization of frozen sections from the surgery, histopathological results were ultimately confirmed.

### BRAF mutation analysis

According to previous researches, the capability to detect BRAF^V600^^E^ mutation from US-guided FNAB cytologic specimens is not inferior to that in postoperative pathologic specimens [21, 22]. The polymerase chain reaction (PCR) conditions and primers for amplifying exon 15 in BRAF containing V600E mutation were established previously. Genomic DNA was extracted from cytologic specimens through the QIAamp DNA FFPE Tissue Kit (QIAGEN) following the manufacturer’s instruction.

### Statistical analysis

Statistical analysis was conducted using SPSS software (ver. 22.0; SPSS Inc., Chicago, IL, USA). Comparison of continuous and categorical variables were analyzed by Student’s t-test and pearson X_2_ or Fisher’s exact test, respectively. Independent predictors for cervical LNM were identified by multivariate logistic analysis. P value less than 0.05 was regarded to be significant.

## Results

### Participant demographics and US features

After the strict inclusion, 112 participants met the criteria and were included for further research. Of included participants, 35 (31.3%) were male and 77 (68.7%) were female. Among them, the mean age was 42.95 years old, and the range of age was from 21 to 66 years old. Briefly, 86 (76.8%) were less than 55 years, and 26 (23.2%) were 55 years and older. 65 participants (58.0%) had cervical CLNM, whereas 47 (42.0%) did not. No distant metastases were found in our research. The average of PTC size was 11.2 mm, and the size range was from 3 to 32 mm. In 55 (49.1%) participants, the tumor size was ≤ 10 mm; and the tumor size was > 10 mm in 57 (50.8%) participants. Multifocal PTCs was observed in 30 (26.8%) cases. 56 (50.0%) were in the left lobe, 49 (43.8%) were in the right lobe and 7 (6.2%) were in the isthmus. Suspicious US features with respect to solid component, marked hypo-/hypo-echogenicity, microcalcifications, irregular/lobulated margins, non-parallel orientation and taller than wide shape were presented in 105 (93.8%), 95 (84.8%), 76 (67.9%), 69 (61.6), 35 (31.3%) and 50 (44.6%) of PTC samples, respectively. Typically, BRAF^V600^^E^ mutation was observed in 56 (50.0%) PTC patients. 22 (19.6%) participants were concomitant Hashimoto’s thyroiditis, and 90 (80.4%) patients were without Hashimoto’s thyroiditis***.***

### Univariate analysis

As demonstrated in Table 1, participants less than 55 years of age have a higher frequency of cervical CLNM than those 55 years or older (86.2% vs 63.8%, *P* = 0.006). Among US characterizes of PTC, tumor diameter > 10 mm (*P* = 0.001) and microcalcification (*P* = 0.007) were remarkably correlated the presence of cervical CLNM. Other suspicious US characterizes, such as solid component, marked hypo-/hypo-echogenicity, irregular/lobulated margins, non-parallel orientation, and taller than wide shape were not related to cervical CLNM in PTC (all P value > 0.05). In addition, concomitant Hashimoto’s thyroiditis was more inclined to be non-cervical CLNM (*P* = 0.008). BRAF^V600^^E^ mutation were more likely to present CLNM positive in PTC patients (*P* = 0.007).

**Table 1:** Clinicopathologic and US features of cervical CLNM in PTC patients. *N* = number of papillary thyroid carcinoma; CLNM = central lymph node metastasis; PTC = papillary thyroid carcinoma

Characteristics	Total PTC with or without cervical LNM(*N* = 112)		P value
	Positive (*N* = 65)	Negative (*N* = 47)	
**Age**	39.82 ± 10.29	47.28 ± 11.44	*P* = 0.006
< 55 years	56 (86.2%)	30 (63.8%)	
≥ 55 years	9 (13.8%)	17 (36.2%)	
**Gender**			*P* = 0.838
Male	21 (32.3%)	14 (29.8%)	
Female	44 (67.7%)	33 (70.2%)	
**Tumor size**	12 ± 4.6 mm	9.8 ± 4.6 mm	*P* = 0.001
> 10 mm	42 (64.6%)	15 (31.9%)	
≤ 10 mm	23 (35.4%)	32 (68.1%)	
**Multifocality**			*P* = 0.832
Multifocal	18 (27.7%)	12 (25.5%)	
Unifocal	47 (72.3%)	35 (74.5%)	
**Location**			*P* = 0.619
Left	35 (53.8%)	21 (44.7%)	
Right	26 (40%)	23 (48.9%)	
Isthmus	4 (6.2%)	3 (6.4%)	
**Internal component**			*P* = 1.000
Solid	61 (93.8%)	44 (93.6%)	
Cystic	4 (6.2%)	3 (6.4%)	
**Marked hypo-/hypo-echogenicity**			*P* = 1.000
Present	55 (84.6%)	40 (85.1%)	
Absent	10 (15.4%)	7 (14.9%)	
**Microcalcification**			*P* = 0.007
Present	51 (78.5%)	25 (53.2%)	
Absent	14 (21.5%)	22 (46.8%)	
**Margin**			*P* = 0.555
Irregular/lobulated	42 (64.6%)	27 (57.4%)	
Regular	23 (35.4%)	20 (42.6%)	
**Orientation**			*P* = 0.838
Non-parallel	21 (32.3%)	14 (29.8%)	
Parallel	44 (67.7%)	33 (70.2%)	
**Taller than wide**			*P* = 0.705
Present	28 (43.1%)	22 (46.8%)	
Absent	37 (56.9%)	25 (53.2%)	
**T stage**			*P* = 0.180
T1	29 (44.6%)	29 (61.7%)	
T2	35 (53.8%)	17 (36.2%)	
T3/T4	1 (1.5%)	1 (2.1%)	
**Hashimoto’s thyroiditis**			*P* = 0.008
Concomitant	7 (10.8%)	15 (31.9%)	
Non-concomitant	58 (89.2%)	32 (68.1%)	
**Internal vascularity**			*P* = 0.846
Present	27 (41.5%)	18 (38.3%)	
Absent	38 (58.5%)	29 (61.7%)	
**BRAF mutation**			*P* = 0.007
Positive	40 (61.5%)	16 (34.0%)	
Negative	25 (38.5%)	31 (66.0%)	

### Multivariate logistic analysis

**Fig. 1:**
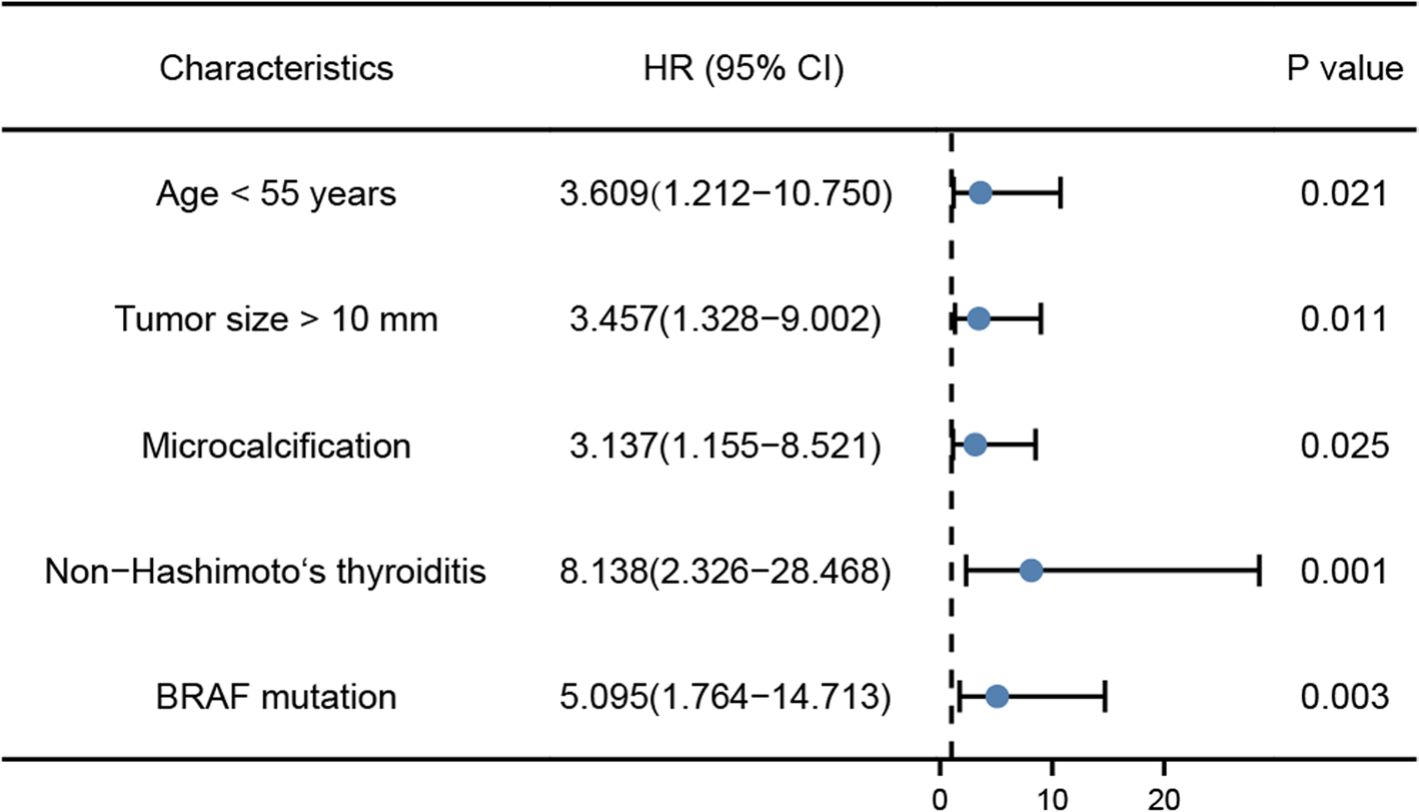
Forest plot of the risk factors of cervical CLNM in PTC patients

**Fig. 2:**
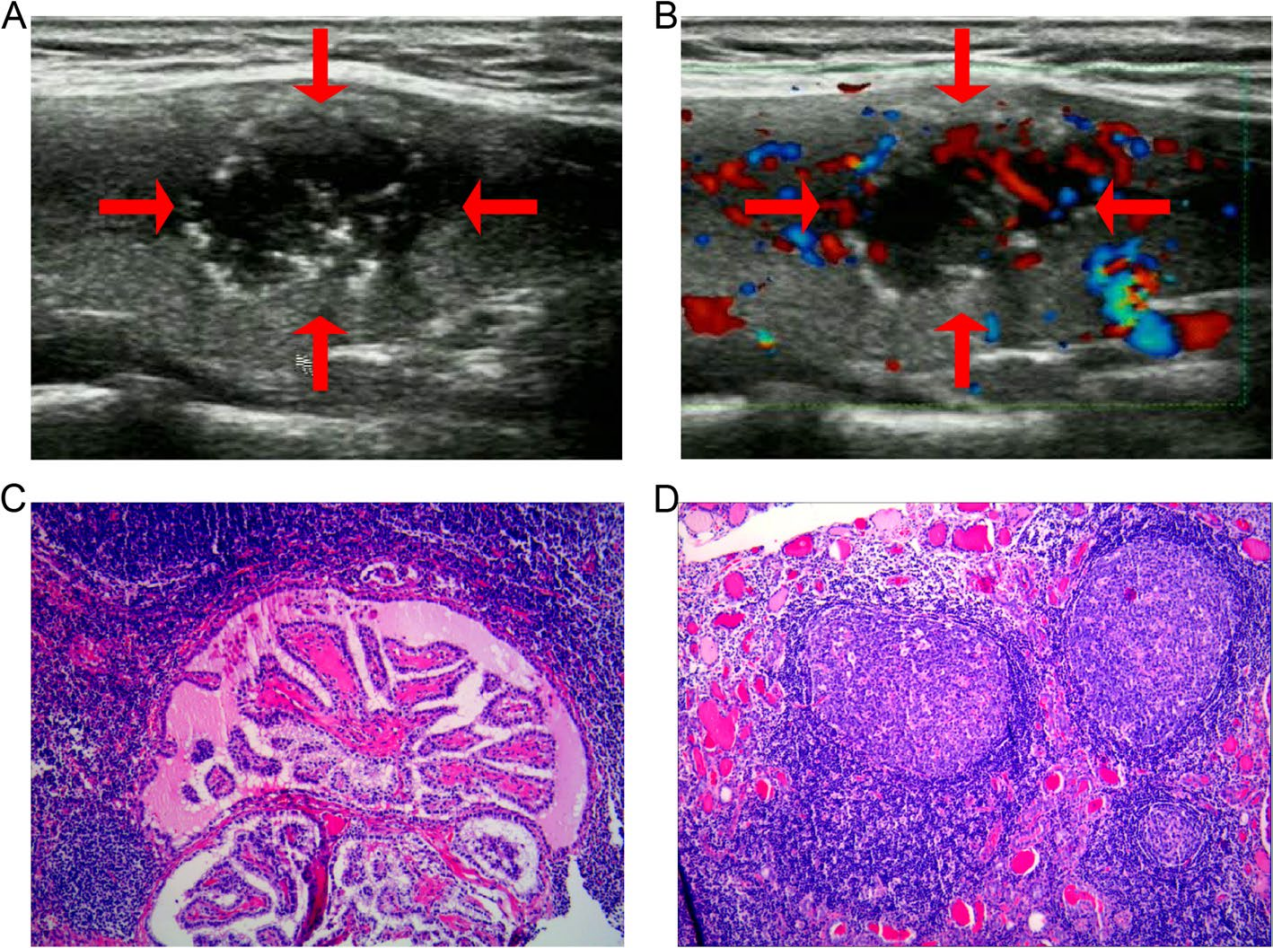
Ultrasonic image showing a 17-mm papillary thyroid cancer in a 22 years-old woman harboring cervical CLNM. **a** The papillary thyroid cancer with tumor size > 10 mm and microcalcification on US. **b** Blood flow is shown on color doppler image. **c** Pathologic examination confirmed the diagnosis of metastatic central lymph node (hematoxylin–eosin stain, × 100). **d** Another PTC participant without cervical CLNM. Pathologic examination confirmed the diagnosis of Hashimoto’s thyroiditis (hematoxylin–eosin stain, × 100)

**Fig. 3:**
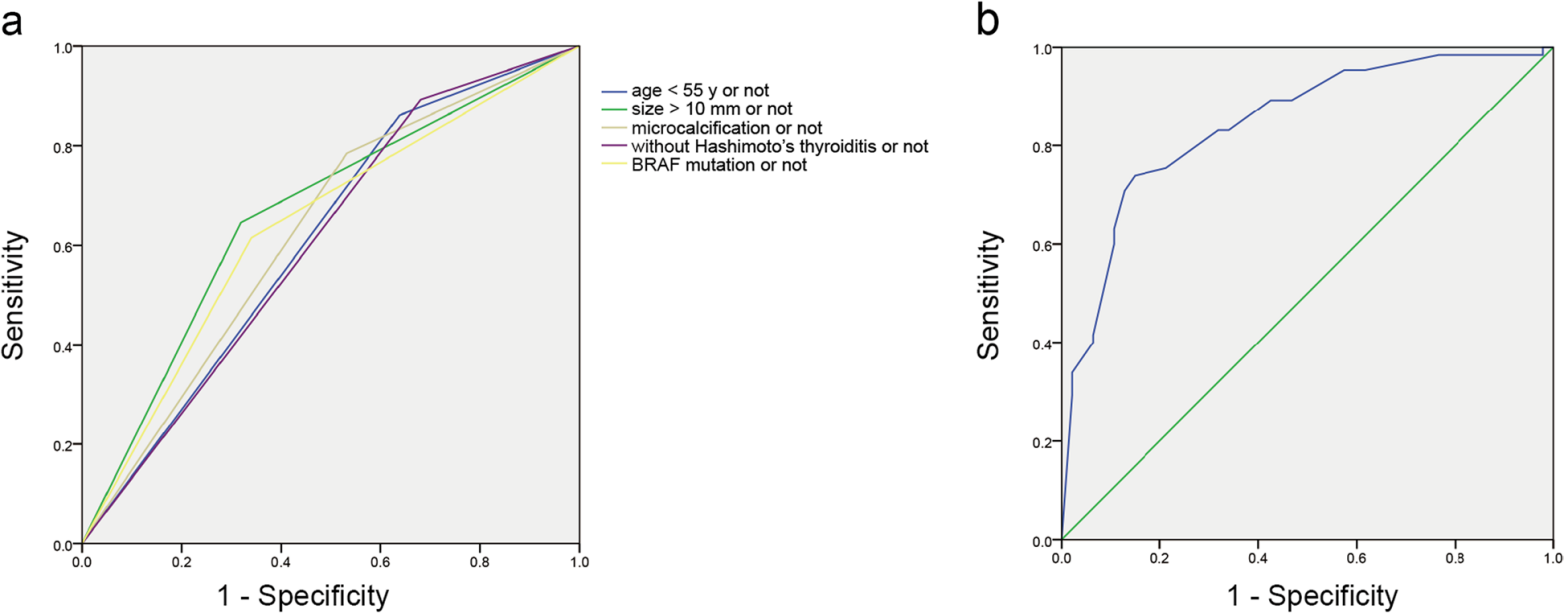
Receiver operating characteristic (ROC) curves of (**a**) age < 55 years old (area under the ROC curve [AUROC] = 0.664), tumor size > 10 mm (AUROC = 0.612), microcalcification (AUROC = 0.626), non-concomitant Hashimoto’s thyroiditis (AUROC = 0.606) and BRAF^V600E^ mutation (AUROC = 0.637), respectively. (**b**) Equation (AUROC = 0.845) for the prediction of cervical CLNM

Moreover, independent risk factors were determined after multivariate analysis (Table 2, Fig. 1). In our research, predictive risk factors, including age patients younger than 55 years of age (OR = 3.609, *P* = 0.021), tumor size > 10 mm (OR = 3.457, *P* = 0.011), microcalcification (OR = 3.137, *P* = 0.025), non-concomitant Hashimoto’s thyroiditis (OR = 8.138, *P* = 0.001), and BRAF^V600^^E^ mutation (OR = 5.095, *P* = 0.003) turned out to be risk predictors for cervical CLMN in participants with PTC (Fig. 2). Then, ROC curves were plotted, and the diagnostic value of the risk factors was discriminative with areas under the ROC curves of 0.612 (95% CI: 0.504–0.720), 0.664 (0.561–0.766), 0.626 (0.520–0.733), 0.606 (0.497–0.714) and 0.637 (0.533–0.742), respectively. Furthermore, their sensitivity and specificity were 86.2% and 36.2%, 64.6% and 68.1%, 78.5% and 46.8%, 89.2% and 31.9%, and 61.5% and 66.0%, respectively (Fig. 3a, Table 3). A multivariate logistic regression equation was established with these independent predictive factors: *P* = 1/1 + expΣ[ -4.366 + 1.284 × (if year < 55y) + 1.241 × (if nodule size > 10 mm) + 1.143 × (if microcalcification in nodule) – 2.097 × (if concomitant Hashimoto’s thyroiditis) + 1.628 × (if BRAF^V600E^ mutation)] (Fig. 3b).

**Table 2:** Multivariate logistic regression analysis in predicting cervical CLNM in PTC patients. CLNM = central lymph node metastasis; PTC = papillary thyroid carcinoma

PTC characteristics	β Coefficient	Odds ratio	95% Confidence Interval	P value
Age < 55 years	1.284	3.609	1.212–10.750	0.021
Tumor size > 10 mm	1.241	3.457	1.328–9.002	0.011
Microcalcification	1.143	3.137	1.155–8.521	0.025
Non-Hashimoto’s thyroiditis	2.097	8.138	2.326–28.468	0.001
BRAF mutation	1.628	5.095	1.764–14.713	0.003

**Table 3:** ROC analysis of the independent factors and equation for predicting cervical CLNM in PTC patients. CI = confidence interval; CLNM = central lymph node metastasis; *N* = number of papillary thyroid carcinoma; PTC = papillary thyroid carcinoma

	Az	95% CI	Cutoff value	Sensitivity	Specificity
Overall (*N* = 112)					
Age < 55y	0.612	0.504–0.720	Age < 55y	0.862	0.362
Tumor size > 10 mm	0.664	0.561–0.766	Tumor size > 10 mm	0.646	0.681
Microcalcification	0.626	0.520–0.733	Microcalcification	0.785	0.468
Non-Hashimoto’s thyroiditis	0.606	0.497–0.714	Non-Hashimoto’s thyroiditis	0.892	0.319
BRAF mutation	0.637	0.533–0.742	BRAF mutation	0.615	0.660
Predictive equation	0.845	0.771–0.918	0.593	0.738	0.851

## Discussion

PTC is recognized as one of the indolent diseases with a satisfactory prognosis, and the 5-year survival rate is nearly to 98% [23, 24]. Despite the death rate is negligible, the recurrent and metastatic PTC may happen in around 30% of PTC patients, which are largely associated with metastasis of cervical lymph nodes [25]. The routine neck dissection in the management for PTC remains a great deal of controversies. Not all cervical CLNM can be detected by US preoperatively, despite US technology is useful in identifying the stage of PTC [26].

US-guided FNAB technology is extensively performed to increase the diagnostic accuracy by cytologic or genomic examination [27]. Furthermore, the ability of BRAF detection in cytology is not inferior to that in pathologic specimens after surgy [28], which can provide us to pre-operatively analyze BARF status from FNAB cytologic specimens. BRAF^V600^^E^ mutation is a somatic mutation that contributes to the transformation of valine to glutamate in the BRAF protein 600-bit codon, the abnormal cellular proliferation, and the malignant tumor formation [29]. Previous research have reported that BRAF^V600E^ mutation is the critical gene of PTC, with about 73% mutation rate [30]. BRAF^V600E^ mutation is associated with poor prognosis, cervical CLNM and recurrence of PTC [16, 31]. In our study, we detected BRAF status from FNAB tissues, and discovered BRAF^V600E^ mutation was a predictive factor for CLNM.

Suspicious US features (size > 10 mm and microcalcification) and age < 55 years were risk predictors for cervical CLMN based on the multivariate logistic analysis. Hashimoto’s thyroiditis was identified as a negatively predictive factor by multivariate logistic analysis. Tumor size is a critical factor estimated for the biological features of PTC, because tumor size can be readily obtained by preoperative US examination [32]. Malignant-looking PTC was more prevalence with extrathyroidal extension, cervical CLNM, and advanced stage than benign-looking PTC, especially when tumor diameter more than 10 mm [33]. Other literatures have covered that tumor size was regarded as an independent predictors for nodal metastasis in PTC patients, which may be explained by the more extensive the PTC, the more proliferative and aggressive [34–36]. Microcalcification is the deposition of calcium salts resulted from vascular and fibrous hyperplasia, indicating the rapid growth of malignant cancer [37]. Thus, once microcalcification can be detected in PTC tissues, the lymph node in cervical scope must be evaluated more cautiously.

Age is a crucial variate in prognosis for patients bearing well-differentiated thyroid diseases. According to up-date-date AJCC guideline, 55 years of age was accustomed to being the cut-off value in staging [38]. Whether age is related to cervical CLNM in PTC patients, the present results are often inconsistent. For example, some reported that cervical CLNM was not related to age [39], while other findings argued that age less than 55 years of age was a predictor for cervical LNM [40, 41]. In this research, the younger age was largely related to a higher odds ratio of cervical CLNM and age younger than 55 years was a risk predictor for cervical CLNM, indicating cervical CLNM should be paid more attention in the younger PTC patients. Also, similar findings have been published by other authors [42, 43].

HT is the most common autoimmune thyroid disease, and the diagnosis of HT was based on the pathology of postoperative specimen in our research. Previous findings have implied a protective effect of HT in PTC patients, which is less associated with ETE, cervical CLNM, advanced stage and recurrence [44, 45]. A review summarized and updated the findings demonstrated that the benefit of HT in thyroid cancer patients depends on the phenotypes of infiltrative lymphocytes [46]. Infiltrative lymphocytes, including B cells, T cells, macrophages, NK cells and Th17 cell, were closely associated with a better outcome in PTC patients [47].

### Limitations

There are still some limitations in our research. Firstly, our study was a retrospective study, and hence, selection bias was unavoidable. Secondly, the US detections were not prospectively standardized, i.e., ideally, every examination followed the same procedure. Thirdly, the disease-specific survival and recurrence of PTC were not analyzed attributed to short follow-up time. Therefore, the future multicenter and prospective research are urgently demanded to estimate the risk factors of nodal metastasis in PTC patients.

## Conclusions

Our comprehensive analysis has determined age < 55 years old, size > 10 mm, microcalcification, non-concomitant Hashimoto’s thyroiditis and BRAF^V600^^E^ mutation are independent predictors for cervical CLNM in PTC patients. BRAF^V600E^ mutation by pre-operative US-FNA technology synergized with clinicopathologic and US characteristics can guide the suitable therapeutic management of PTC. Overall, our research is expected to estimate PTC aggressiveness, thereby avoiding overtreatment and undertreatment, such as unnecessary prophylactic CLND, and providing emerging strategies for precise medicine for individual patients.

## Data Availability

All data generated or analyzed during this study are included in this published article.
